# Generation of a conditional knockout allele for the NFAT5 gene in mice

**DOI:** 10.3389/fphys.2014.00507

**Published:** 2015-01-05

**Authors:** Christoph Küper, Franz-Xaver Beck, Wolfgang Neuhofer

**Affiliations:** ^1^Department of Physiology, University of MunichMunich, Germany; ^2^Medical Clinic V, University Hospital Mannheim, University of HeidelbergMannheim, Germany

**Keywords:** NFAT5, knockout mouse model, TonEBP, osmosensing, kidney physiology

## Abstract

The osmosensitive transcription factor nuclear factor of activated T-cells 5 (NFAT5), also known as tonicity enhancer element binding protein (TonEBP) plays a crucial role in protection of renal medullary cells against hyperosmotic stress, urinary concentration, the adaptive immune response, and other physiological systems. Since it is also important for development, conventional homozygous-null mutations result in perinatal death, which hinders the analysis of NFAT5 function in specific tissues *in vivo*. Here we describe the generation of mice with a conditional-null allele, in which *loxP* sites are inserted around exon 4. Mice harboring the floxed allele (*NFAT5^flx^*) were mated to a strain expressing a tamoxifen-inducible derivative of the *Cre*-recombinase (*Cre*^+^) under the control of the ubiqitinC promoter. The resultant homozygous conditional knockout mice (*Cre*^+^ NFAT5^*flx/flx*^) are viable, fertile, and show normal expression of NFAT5 and NFAT5 target genes, indicating that the conditional alleles retain their wild-type function. Induction of *Cre*-mediated recombination by administration of tamoxifen in 8-week-old mice resulted in a decrease in NFAT5 expression of about 70–90% in all tested tissues (renal cortex, renal outer medulla, renal inner medulla, heart, lung, spleen, skeletal muscle). Accordingly, the expression of the NFAT5 target genes aldose reductase and heat shock protein 70 in the renal medulla was also significantly decreased. Mice harboring this conditional knockout allele should be useful in future studies for gaining a better understanding of tissue and cell-type specific functions of NFAT5 in adult animals under physiological and pathophysiological conditions.

## Introduction

The osmosensitive transcription factor tonicity-responsive enhancer binding protein (TonEBP) was first identified in renal medullary cells. Based on its homology to nuclear factors of activated T cells (NFAT) 1–4, TonEBP is also referred to as NFAT5. NFAT5 drives the expression of various genes that are important for the function of the renal medulla. On the one hand, NFAT5 regulates the expression of central components of the urinary concentrating mechanism, including the kidney-specific ClC-K1 chloride channels and their functional subunit barttin (Küper et al., 2012b), and UT-A1 urea transporters (Nakayama et al., 2000). Accordingly, NFAT5 is a prerequisite for building up the cortico-medullary osmotic gradient by the interstitial accumulation of NaCl and urea in the renal papilla. NFAT5 is further indispensible for the production of concentrated urine during antidiuresis by virtue of its regulation of the transcription of aquaporin-2 (AQP-2) water channels in collecting duct principle cells (Hasler et al., 2006; Hasler, 2011). NFAT5 is, moreover, essential for the osmoadaptation of cells in the renal medulla. In this kidney region, NFAT5 stimulates the expression of transporters and biosynthetic enzymes for cellular accumulation of organic osmolytes. These include the Na-*myo*-inositol cotransporter (SMIT) (Miyakawa et al., 1999), the Na-Cl-betaine cotransporter (BGT1) (Miyakawa et al., 1998), the Na-Cl-taurine cotransporter (TauT) (Ito et al., 2006), aldose reductase (AR) (Nadkarni et al., 1999), specific heat shock proteins (Woo et al., 2002), and others. The intracellular accumulation of organic osmolytes and heat shock protein 70 (HSP70) protect cells from the adverse effects of hypertonicity.

In past years, it has become increasingly apparent that, in addition to its role in the urinary concentration mechanism and in protection against hyperosmotic stress, NFAT5 is also involved in diverse other physiological and pathophysiological processes. NFAT5 drives for instance the osmotic stress-induced expression of proinflammatory cytokines LT-β, Il-6, and TNF-α in macrophages (Lopez-Rodriguez et al., 2001; Buxade et al., 2012) and of the chemokine MCP-1 (CCL2) in epithelial cells during inflammatory processes (Kojima et al., 2010; Roth et al., 2010; Küper et al., 2012a), indicating that NFAT5 enhances the proinflammatory immune response under pathophysiological conditions associated with hyperosmotic stress. In breast and colon carcinoma, Integrin-mediated activation of NFAT5 stimulates the expression of S100A4, thereby promoting metastatic activity of tumor cells (Jauliac et al., 2002; Chen and O'Connor, 2005; Chen et al., 2009, 2011). Knockdown of NFAT5 in T-lymphocytes challenged with osmotic stress results in downregulation of cyclins E1, A2, and B1, which is associated with cell cycle arrest (Drews-Elger et al., 2009). In skeletal muscle cells, NFAT5 regulates expression of Cyr61, thereby controlling migration and differentiation of myoblasts (O'Connor et al., 2007). In the placenta, NFAT5 stimulates the expression of SMIT, AR, and HSP70 (Arroyo et al., 2009, 2012; Park et al., 2014).

Most of these findings have been derived from cell culture experiments. Conventional NFAT5 knockout mice show high perinatal lethality due to impaired renal (Lopez-Rodriguez et al., 2004) and heart (Mak et al., 2011) development. Furthermore, these animals show an impaired immune response (Trama et al., 2002; Go et al., 2004). Thus, conventional NFAT5 knockout mice are of limited use for analysis of NFAT5 function in adult animals. The goal of the present study was to create a conditional NFAT5 knockout allele to overcome the problems of conventional NFAT5 knockout mice. For this purpose, we constructed a NFAT5 allele, in which exon 4 is flanked by *loxP* sequences (NFAT5^*flx*^). *Cre*-mediated recombination of this allele leads to knockdown of NFAT5. By mating NFAT5^*flx*^ mice with a mouse strain expressing a tamoxifen-inducible *Cre* recombinase, we generated a mouse strain in which NFAT5 can be knocked down in adult animals by tamoxifen administration.

## Materials and methods

### Construction of a conditional targeting vector for NFAT5

The conditional NFAT5 allele was created in collaboration with Ingenious Targeting Laboratory, Inc., (Stony Brook, NY, USA). A 8.26-Kb region from a positively identified C57BL/6 BAC clone (RP23: 187J8) was used to construct the targeting vector. The region was subcloned into a pSP72 vector (Promega, Madison, WI, USA) containing an ampicillin selection cassette. The short homology arm (SA) of the region was designed such that it extended about 2.45 kb 3′ to exon 4 of the NFAT5 gene. The long homology arm (LA) ended 5′ to exon 4, and was 5.25 kb long. A pGK-gb2 *loxP*/*frt Neo* cassette was inserted 202 bp downstream from exon 4. The *Neo* cassette contained the promoter of the mouse phosphoglucokinase gene, a neomycin resistance gene, and a synthetic polyadenylation sequence, and was flanked by *frt* sites for later excision by *Flp* recombinase. The cassette also contained two *loxP* sites, one within the *frt* sites, and another one at the 3′ end of the cassette, outside the *frt* sites. An additional single *loxP* site (third *loxP* site), containing engineered KpnI, EcoRI, MscI and StuI sites for southern blot analysis, was inserted 166 bp upstream from exon 4. The length of the target region was 561 bp and included exon 4; the total size of the targeting construct (including vector backbone and *Neo* cassette) was 12.4 kb. The targeting vector was confirmed by restriction analysis after each modification step. P6 and T7 primers (see Table 1) anneal to the vector sequence and read into the 5′ and 3′ ends of the BAC sub clone. N1 and N2 primers anneal to the 5′ and 3′ ends of the *loxP*/*frt Neo* cassette and sequence the SA and LA, respectively.

**Table 1:** **Oligonucleotides used in this study**.

**Name**	**Sequence**	**Purpose**
P6	5′-GAG TGC ACC ATA TGG ACA TAT TGT C-3′	Gene targeting
T7	5′-CGA TAA GCC AGG TTA ACC TGC ATT A-3′	Gene targeting
N1	5′-TGC GAG GCC AGA GGC CAC TTG TGT AGC-3′	Gene targeting
N2	5′-TTC CTC GTG CTT TAC GGT ATC G-3′	Gene targeting
loxP	5′-TGA GAA ACA TGT ATG TGG GGC ATG-3′	Gene targeting
LOX2	5′-GTA ACC ATG ATT AGT CTT TTA GCT TTA TG-3′	Gene targeting, genotyping
SDL2	5′-GTT CTG AGA ATC CAA AGC ACA AC-3′	Gene targeting, genotyping
NDEL1	5′-GTT GTG CTT TGG ATT CTC AGA AC-3′	Gene targeting
NDEL2	5′-CTT CTA CCC TTC TAT TTC AGG AAG-3′	Gene targeting
A1	5′-CAC AAA TAC CTG CAA CAC CAG TGG-3′	Gene targeting
A2	5′-ACG CCA GTG TCA TGT TGT TG-3′	Gene targeting
A3	5′-GGA AAC AGA TAA CAT GCA TCA TAA ACA-3′	Gene targeting
AT1	5′-AAAT CGT AGG CTA GTA CTC CAC CC-3′	Gene targeting
AT2	5′-AGC TCA GGA AAA GCT TCC TG-3′	Gene targeting
F3	5′-GCA TAA GCT TGG ATC CGT TCT TCG GAC-3′	Gene targeting
F7	5′-GGA ACT TCG CTA GAC TAG TAC GCG TG-3′	Gene targeting
LUNI	5′-GCA TCG CCT TCT ATC GCC TTC TTG-3′	Gene targeting
PB1.1	5′-TTT TGT GGC TAA GCA CAGT CCC-3′	Southern blot probe
PB1.2	5′-CAT ACT GCA GCT CTG CTC AGA TCC-3′	Southern blot probe
PB2.1	5′-TGA CTG CCC TCA ACA GTT CAT TTG-3′	Southern blot probe
PB2.2	5′-ATT CAG GAT CTG CTA CCA CCA CTG-3′	Southern blot probe
Cre320 sense	5′-GAA CCT GAT GGA CAT GTT CA-3′	Genotyping
Cre320 anti	5′-AGT GCG TTC GAA CGC TAG AGC CTG T-3′	Genotyping
NFAT5 sense	5′-AAC ATT GGA CAG CCA AAA GG-3′	qRT-PCR analysis
NFAT5 anti	5′-GCA ACA CCA CTG GTT CAT TA-3′	qRT-PCR analysis
AR sense	5′-CTT AAA ATA TAA GCC TGC GGT GA-3′	qRT-PCR analysis
AR anti	5′-GCC TTT GCT GTG GCA GTA TT-3′	qRT-PCR analysis
BGT-1 sense	5′-GGC TCC TTT TGG TCA CAG AG-3′	qRT-PCR analysis
BGT-1 anti	5′-GCT GGA GGC GTA GTA GTC AAA-3′	qRT-PCR analysis
HSP70 sense	5′-TGA GTC CCA CAC TCT CAC CA-3′	qRT-PCR analysis
HSP70 anti	5′-CTG TGG GTG AAG CTG TTA AGG-3′	qRT-PCR analysis
β-Actin sense	5′-CTA AGG CCA ACC GTG AAA AG-3′	qRT-PCR analysis
β-Actin anti	5′-ACC AGA GGC ATA CAG GGA CA-3′	qRT-PCR analysis

### Targeted ES cells and generation of chimeric mice

The targeting vector (10 μg) was linearized by NotI and then transfected by electroporation of BA1(C57BL/6x129/SvEv) hybrid embryonic stem (ES) cells. After selection with G418 antibiotic, surviving clones were expanded for PCR analysis to identify recombinant ES clones. ES cell colonies were first screened by PCR, using primers A1 or A2, binding downstream from the SA, and the primer F3, binding within the *Neo* cassette. Size of the resulting PCR products was 2.57 or 2.73 kb, respectively. A control PCR reaction was performed using the 3′-primer AT1 and the 5′-primer AT2, which bind downstream from the *Neo* cassette within the SA. The size of the resulting PCR product was 2.25 kb.

### PCR confirmation of the third *loxP* site

PCR was performed on SA positive clones to detect presence of the third *loxP* site (5′-region of exon 4) using the LOX2 and SDL2 primers. This reaction amplifies a 334-bp wild-type product. The presence of a second PCR product 392 bp in size indicates a positive *loxP* PCR. Positive clones were sequenced to confirm sequence integrity and presence of the third *loxP* site using the SDL2 primer.

### Reconfirmation of expanded clones by Southern blot

Four individual clones that had passed all controls were reconfirmed by Southern blot analysis. DNA was digested with StuI, and electrophoretically separated on a 0.8% agarose gel. After transfer to a nylon membrane, the digested DNA was hybridized with a probe targeted against the 5′ external region of the LA, resulting in detection of two bands at 6.7 kb (targeted allele), and 9.5 kb (wild-type allele). Positive clones were further confirmed by Southern blot analysis using an internal probe. DNA was digested with PvuII, and electrophoretically separated. After transfer to a nylon membrane, the digested DNA was hybridized with a probe targeted against the 3′ internal region of the SA, resulting in detection of two bands at 3.5 kb (targeted allele), and 9.2 kb (wild-type allele). DNA from C57Bl/6 (B6), 129/SvEv (129), and BA1 (C57Bl/6 × 129/SvEv) (Hybrid) mouse strains were used as wild-type controls.

Targeted iTL BA1 hybrid embryonic stem cells were microinjected into C57BL/6 blastocysts. Resulting chimeras with a high percentage of agouti coat color were mated to wild-type C57BL/6 homozygous *Flp* mice to remove the *Neo* cassette. Tail DNA was analyzed from pups with agouti or black coat color. Presence of the third *loxP* site was screened by PCR using LOX2 and SDL2 primers; presence and correct integration of the third *loxP* site was also confirmed by sequencing using LOX2 primer. Presence and confirmation of the SA was screened using primers A3 and F3, resulting in a 2.5-kb amplification product. Primers NDEL1 and NDEL2 were used to screen the genomic DNA for deletion of the *Neo* cassette. This reaction amplifies a 492-bp wild-type product, the presence of a second 675 bp PCR product indicates a targeted allele with deletion of the *Neo* cassette. Presence of the *Neo* cassette resulted in no amplification product because of its too big size. Finally, heterozygous F1 mice with confirmed *loxP*-sites and *Neo* deletion were back-crossed with C57/BL6 mice for 10 generations to obtain heterozygous mice with a >99.9% C57/BL6 background.

### Knockdown of NFAT5

Mice carrying an NFAT5 allele with a *loxP*-flanked exon 4 (NFAT5^*flx*^) were mated to the strain B6.Cg-Tg(UBC-cre/ERT2)1Ejb/J (Jackson Laboratory, Bar Harbor, ME, USA) harboring a *Cre*-ERT2 transgene under control of the ubiquitinC promoter. *Cre*^+^ NFAT5^*flx*/+^ mice were interbred, and *Cre*^+^ NFAT5^*flx/flx*^ mice were used for knockdown of NFAT5. To induce the knockdown, 6-week-old mice were fed a tamoxifen-containing (400 mg/kg) diet (*Cre*-active 400, Lasvendi, Soest, Germany) for 4 weeks. Subsequently, mice were sacrificed, and various tissues (kidney (cortex, outer medulla, inner medulla), liver, heart, spleen, lung) were snap frozen in liquid nitrogen and later analyzed for expression of NFAT5 and NFAT5 target genes. All animal experiments were in accordance with the institutional and the national guidelines and regulations. The experimental procedures were approved by the institutional animal health and care committee.

### qRT-PCR analysis

For determination of NFAT5, AR, HSP70, BGT-1, and β-actin mRNA expression levels, tissues were homogenized in TRIFAST Reagent (Peqlab, Erlangen, Germany), and RNA was isolated as recommended by the manufacturer. Oligonucleotides used in this experiment are listed in Table 1. Experiments were carried out on a Roche LightCycler 480, using the SensiMix SYBR One-Step Kit (Bioline, Luckenwalde, Germany) according to the manufacturer's recommendations. Specificity of PCR product formation was confirmed by monitoring melting point analysis and by agarose gel electrophoresis.

### Immunoblot analysis

Antibodies against NFAT5 and actin were obtained from Sigma (Deisenhofen, Germany); antibody against HSP70 was obtained from Stressgen (Victoria, Canada); antibody against rat lens aldose reductase was a kind gift from Peter F. Kador. Tissues were homogenized in 8 M urea buffer. Aliquots (5–30 μg protein) were subjected to sodium dodecylsulphate polyacrylamide gel-electrophoresis (SDS-PAGE) and blotted onto nitrocellulose membranes (Amersham Pharmacia Biotech, Little Chalfont, Bucks., UK). Non-specific binding sites were blocked with 5% non-fat dry milk in PBS containing 0.1% Tween-20 (PBS-T) at room temperature for 1 h. Samples were incubated with primary antibodies in PBS-T containing 5% non-fat dry milk over night at 4°C. Subsequently, the blots were washed 3 times with PBS-T for 5 min each. Subsequently, the membranes were incubated with appropriate secondary antibody at room temperature for 1 h in PBS-T containing 5% non-fat dry milk. After washing with PBS-T 3 times for 5 min each, immunocomplexes were visualized by enhanced chemiluminescence (Pierce, Rockford, IL, USA).

### Statistical analyses

Data are expressed as means ± SEM. The significance of differences between the means was assessed by Student's *t*-test. *P* < 0.05 was regarded as significant. All experiments were performed at least three times and representative results are shown.

## Results and discussion

**Figure 1:**
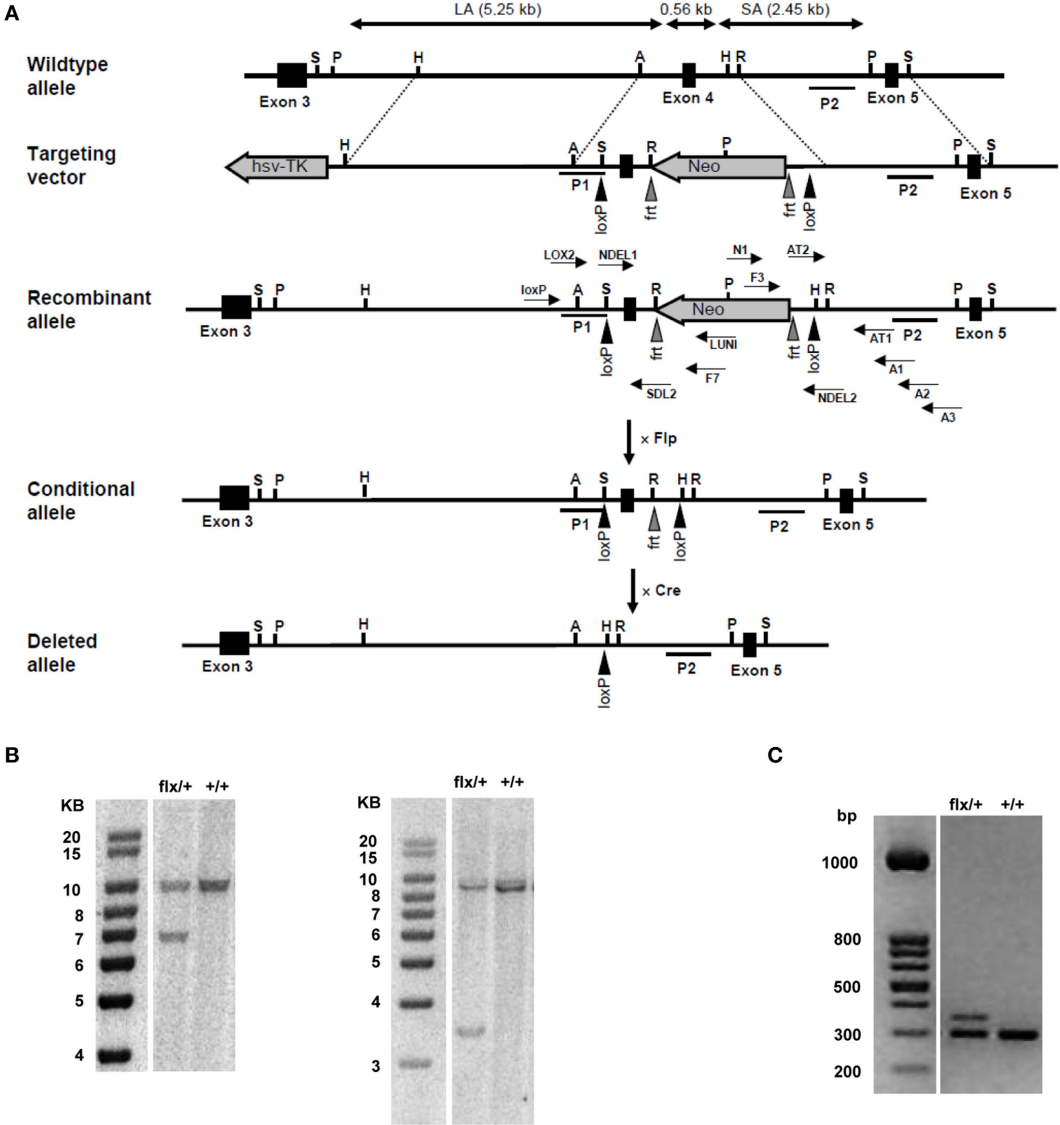
**Generation of NFAT5 conditional null allele. (A)** Schematic figures of the wild-type NFAT5 allele, the targeting vector, and the targeted NFAT5^*neo-flx*^, NFAT5^*flx*^, and NFAT5^Δ^ alleles. Exons are represented by open boxes in which the exon number is indicated. Black triangles denote *loxP* sites; gray triangles denote *frt* sites. The 5′ homologous arm of the targeting vector is ~ 5.25 kb, the 3′ homologous arm ~ 2.45 kb. P1 and P2 denote the location of the 5′- and the 3′- external probe used for Southern blot analysis. Arrows indicate the position of the primers used for gene targeting and genotyping. NFAT5^*neo-flx*^ mice were mated with *Flp* mice to generate NFAT5^*flx*^ mice. NFAT5^Δ^ mice were generated by crossing NFAT5^*flx*^ mice with transgenic *Cre* mice and subsequent tamoxifen administration to targeted offspring. **(B)** Southern blot analysis of NFAT5^*neo-flx*/+^ genomic DNA. 5′- and 3′- external probes were used to detect the presence of the LA (5.25 kb) and the SA (2.45 kb) of the targeted NFAT5 fragment. **(C)** Genotypes of mice were identified using genomic DNA prepared from tail biopsies. Primers LOX2 and SDL2 were used to distinguish the targeted allele (392 bp) from the untargeted wild-type allele (334 bp).

The murine NFAT5 gene is located on the plus (+) strand of chromosome 8, spans about 86 kb, and is divided into 16 exons. To generate a functional NFAT5-null allele in mice, exon 4 was chosen as target for conditional deletion by insertion of flanking *loxP* sites. Exon 4 shares 100% sequence homology with the human NFAT5 exon 4, the perfect homology indicating a highly conserved function for the protein sequence encoded by this exon; additionally, deletion of exon 4 results in a frameshift between exons 3 and 5. To generate the conditional NFAT5-null (NFAT5^*flx*^) allele in mice, a genomic DNA fragment containing the relevant region was isolated from the murine BAC library; a *loxP* sequence was inserted 167 bp upstream from exon 4, and an frt-flanked *Neo* expression cassette containing two *loxP* sites was inserted 202 bp downstream from exon 4 to enable positive selection (Figure 1A). The resulting target vector was transfected into TL1 mouse embryonic stem (ES); then, the ES cell colonies were selected with G418, screened via PCR and Southern blot. Chimeric mice were generated by microinjecting NFAT5^*flx-neo*/+^ ES cells (Figure 1B) into C57BL/6 blastocysts, and germline transmission was verified via obtaining F1 NFAT5^*flx-neo*/+.^offspring from crossing chimeric and C57/Bl6 mice. The F1 NFAT5^*flx-neo*/+^ mice were crossed with *Flp* mice to remove the *Neo* cassette in the germ line and the NFAT5^*flx*/+^ offspring then backcrossed with C57/Bl6 wild-type mice over 10 generations to obtain mice with >99.9% C57/Bl6 background. Thereafter, NFAT5^*flx*/+^ mice were interbred, and the offspring were genotyped via PCR (Figure 1C). The homozygous NFAT5^*flx/flx*^ mice appeared normal, were fertile, and were born in the expected Mendelian ratio. NFAT5 expression, as analyzed by qRT-PCR and immunoblotting, was comparable to C57/Bl6 wild-type mice (data not shown).

Next, homozygous NFAT5^*flx/flx*^ mice were mated to B6.Cg-Tg(UBC-cre/ERT2)1Ejb/J mice. This strain has strong tamoxifen-inducible *Cre* activity in all reported tissue types. Expression of the *cre*/ERT2 transgene is under control of the ubiquitin C promoter. The *Cre*-ERT2 fusion protein consists of *Cre* recombinase fused to a triple mutant form of the human estrogen receptor, which does not bind its natural ligand (17β-estradiol) at physiological concentrations but will bind the synthetic estrogen receptor ligand tamoxifen. Restricted to the cytoplasm, *Cre*-ERT2 can only gain access to the nucleus after exposure to tamoxifen, where *Cre*-mediated recombination will delete *loxP*-flanked sequences from the genome. The offspring from this mating were genotyped for the presence of the *cre* transgene and the NFAT5^*flx*^ allele, and interbred at the age of 8 weeks. Again, the offspring from this mating were genotyped, and *cre*-positive, homozygous NFAT5^*flx/flx*^ mice were used for knockdown of NFAT5.

**Figure 2:**
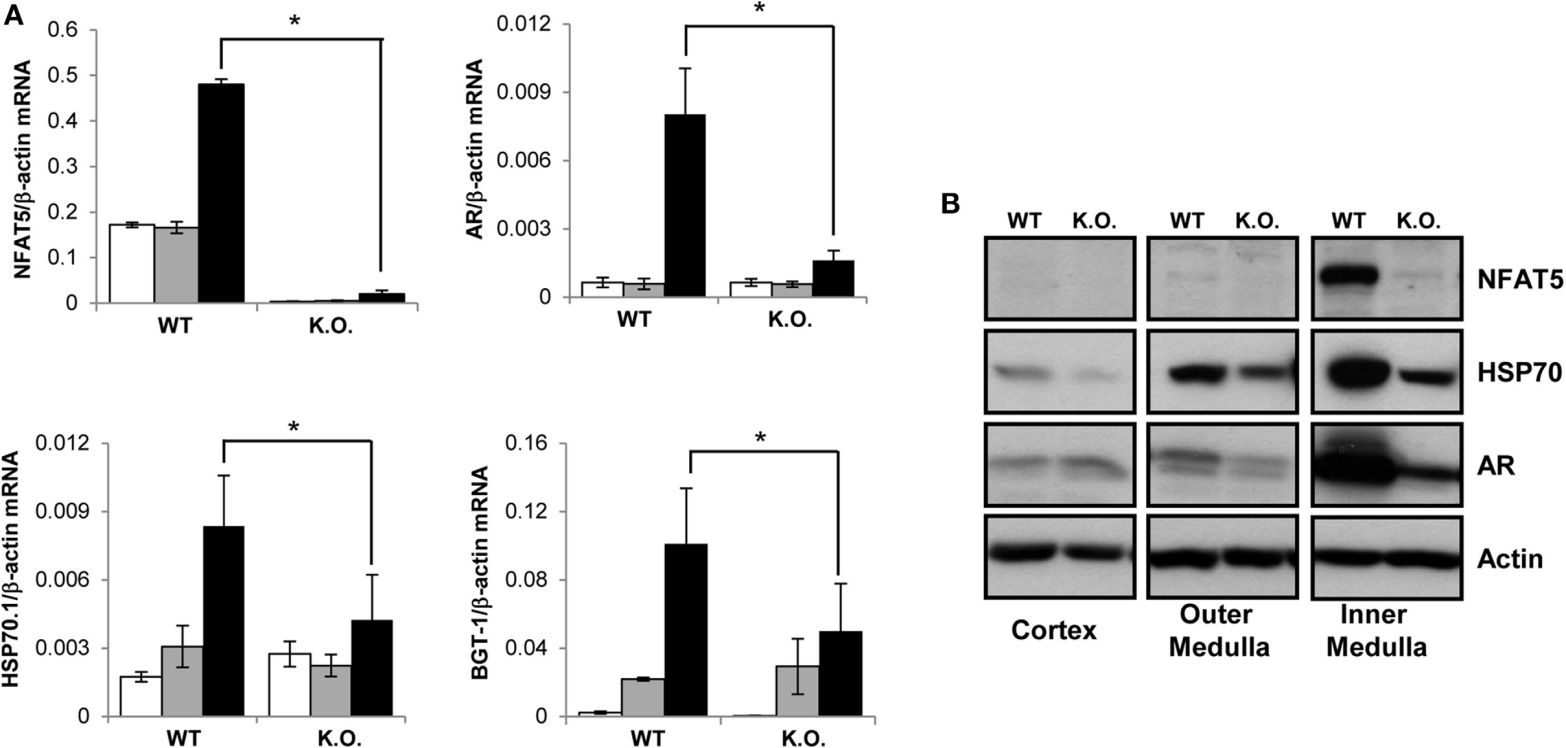
**Expression of NFAT5 and NFAT5 target genes in the kidney**. *Cre*^+^ NFAT5^*flx/flx*^ mice (or *Cre*^+^ NFAT5^+/+^ mice, as control) were fed a tamoxifen-containing diet for 4 weeks to induce *Cre*-mediated excision of exon 4 of the NFAT5 gene. Subsequently, animals were sacrificed and tissue samples obtained from the renal cortex, outer medulla and inner medulla. **(A)** mRNA from renal cortex (☐), outer medulla (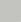) and inner medulla (■) was tested for transcription of NFAT5 and NFAT5 target genes AR, BGT-1 and HSP70 by semiquantitative RT-PCR. Relative mRNA abundance was normalized to that of β-actin to correct for differences in RNA input. Data are means ± SEM for *n* = 4; ^*^P < 0.05. **(B)** Protein samples from renal cortex, outer medulla and inner medulla were tested for protein expression of NFAT5 and NFAT5 target genes AR and HSP70 by Western blot. To demonstrate comparable protein loading, the blots were also probed for actin. Representative blots from at least 4 independent experiments are shown.

**Figure 3:**
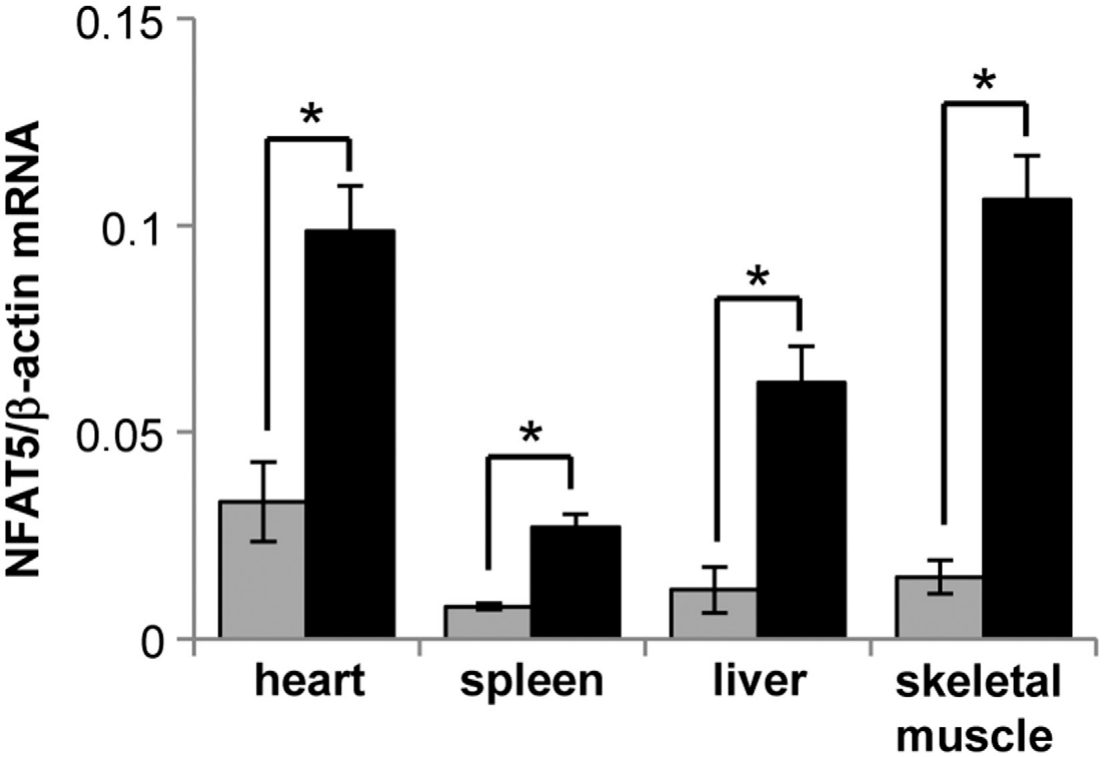
**Expression of NFAT5 in various tissues of conditional knockout mice**. *Cre*^+^ NFAT5^*flx/flx*^ mice (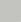) or *Cre*^+^ NFAT5^+/+^ mice (■; as control) were fed a tamoxifen-containing diet for 4 weeks to induce *Cre*-mediated excision of exon 4 of the NFAT5 gene. Subsequently, tissue samples from heart, liver, spleen, and skeletal muscle, were taken and expression of NFAT5 was determined by semiquantitative qRT-PCR. Relative mRNA abundance was normalized to that of β-actin to correct for differences in RNA input. Data are means ± SEM for *n* = 4; ^*^P < 0.05.

To induce the knockdown, *Cre*^+^ NFAT5^*flx/flx*^ mice (or *Cre*^+^ NFAT5^+/+^ mice as control) were fed a tamoxifen-containing diet for 4 weeks. Subsequently, the animals were sacrificed and tissue samples prepared from liver, spleen, heart, skeletal muscle, and kidney Kidney tissue samples were subdivided into cortical, outer medullary, and inner medullary samples. Renal expression of NFAT5 and the designated NFAT5 target genes AR, HSP70, and BGT-1 was tested by semiquantitative RT-PCR (Figure 2A) and immunoblot (Figure 2B). Highest expression of NFAT5 and NFAT5 target genes in control animals was found in inner medullary tissue samples, as expected. In knockout animals, NFAT5 expression was almost completely abolished in all renal tissue sections whilst expression of NFAT5 target genes was reduced significantly in the inner medulla. Expression in cortical and outer medullary sections was largely unaffected. These results indicate that *Cre*-mediated recombination of the NFAT5^*flx*^ allele indeed leads to a knockdown of NFAT5 and under the tested conditions (H_2_O *ad libitum*), regulation of osmoprotective target genes by NFAT5 occurs predominantly in the inner medulla, which is in accordance with previous studies (Lopez-Rodriguez et al., 2004). NFAT5 expression in tissue samples of heart, liver, spleen, and skeletal muscle was tested by qRT-PCR (Figure 3), and expression levels were significantly decreased to 15–25% in NFAT5 knockout animals (compared with 100% in control animals).

In the past, there have been attempts to analyze NFAT5 function *in vivo* by generation of conventional NFAT5 knockout mice. These experiments clearly demonstrated the importance of NFAT5 for embryonic development, however, there are severe limitations on the use of these animals for defining the functions of NFAT5 in adult animals. Conventional NFAT5^−/−^ animals show high embryonic and perinatal lethality. Until E14.5 no significant deviation from the expected Mendelian ratios can be observed in the number of NFAT5^−/−^ embryos, while only 50% of the expected number is obtained at E17.5, and only 3% at P21 (Lopez-Rodriguez et al., 2004). NFAT5^−/−^ embryos show peripheral edema, probably due to abnormal heart development as indicated by reduced cell density of the myocardium and thinner ventricular wall (Mak et al., 2011). The few animals that reach P21 show congenital growth retardation and suffer from renal atrophy, probably due to decreased expression of NFAT5 target genes like AR, HSP70, BGT-1, and SMIT during kidney development (Lopez-Rodriguez et al., 2004). Hence, the kidney is already severely damaged when the animals reach adulthood, and thus research on NFAT5 function in the adult kidney, e.g., its role in the urinary concentration mechanism, is severely restricted in conventional NFAT5^−/−^ mice. Moreover, these animals develop an immune defect due to lymphoid hypocellularity and impaired antigen-specific antibody responses (Go et al., 2004). Mice that express a dominant-negative derivative of NFAT5 exhibit a 25% reduction of CD4+ T-cells and a 50% reduction of CD8+ T-cells (Trama et al., 2002).

Generally, the fact that NFAT5 has multiple physiological and pathophysiological functions indicates that a conventional knockdown is an inappropriate model for investigations on NFAT5 function in a specific cell type or tissue *in vivo*, since systemic NFAT5 knockdown may generate unspecific side effects with unpredictable impact on the tissue of interest. Modern *Cre-Lox* recombination technology would appear to be an appropriate means of overcoming this problem. Today, multiple mouse strains are available, which express constitutively active or inducible *Cre* recombinase with high specificity in individual cell types and tissues, or at various developmental stages. Thus, combination of such restricted *Cre* expression with the NFAT5^*flx*^ allele described in the present study will provide a useful tool for characterizing the tissue-type and cell type-specific functions of NFAT5 in many developmental processes and diseases, as well as physiological functions in adult animals, without the bias produced by unspecific side effects of systemic knockdown.

## Conclusions

Taken together, the results indicate that we have been able to generate a conditional NFAT5-null mutant mouse line. The NFAT5^*flx*^ allele retains the function of the wild-type gene, and inactive NFAT5-null allele can be induced via *Cre*-mediated recombination. This will allow the study of NFAT5 function in specific cell types and tissues *in vivo*.

### Conflict of interest statement

The authors declare that the research was conducted in the absence of any commercial or financial relationships that could be construed as a potential conflict of interest.
